# The prevalence of nutritional anemia in pregnancy in an east Anatolian province, Turkey

**DOI:** 10.1186/1471-2458-10-329

**Published:** 2010-06-10

**Authors:** Leyla Karaoglu, Erkan Pehlivan, Mucahit Egri, Cihan Deprem, Gulsen Gunes, Metin F Genc, Ismail Temel

**Affiliations:** 1 Public Health Department, Medical School, Rize University, Rize, Turkey; 2 Public Health Department, Medical School, Inonu University, Malatya, Turkey; 3 Public Health Department, Medical School, Gaziosmanpaşa University, Tokat, Turkey; 4 Enfectious Diseases Department, Ankara Health Directorate, T.R.Ministry Of Health, Ankara, Turkey; 5 Department of Biochemistry, Medical School, Inonu University, Malatya, Turkey

## Abstract

**Background:**

Anemia is considered a severe public health problem by World Health Organization when anemia prevalence is equal to or greater than 40% in the population. The purpose of this study was to determine the anemia prevalence with the associated factors in pregnant women and to determine the serum iron, folate and B12 vitamin status in anaemic pregnants in Malatya province.

**Methods:**

This is a cross-sectional survey. A multi-sage stratified probability-proportional-to-size cluster sampling methodology was used. A total of 823 pregnant women from sixty clusters were studied. Women were administered a questionnaire related with the subject and blood samples were drawn. Total blood count was performed within four hours and serum iron, folate and B12 vitamin were studied after storing sera at -20 C for six months.

**Results:**

Anemia prevalence was 27.1% (Hb < 11.0 gr/dl). Having four or more living children (OR = 2.2), being at the third trimester (OR = 2.3) and having a low family income (OR = 1.6) were determined as the independent predictors of anemia in pregnancy. Anemia was also associated with soil eating (PICA) in the univariate analysis (p < 0.05). Of anaemic women, 50.0% had a transferrin saturation less than 10% indicating iron deficiency, 34.5% were deficient in B12 vitamin and 71.7% were deficient in folate. Most of the anemias were normocytic-normochromic (56.5%) indicating mixed anemia.

**Conclusions:**

In Malatya, for pregnant women anemia was a moderate public health problem. Coexisting of iron, folate and B vitamin deficiencies was observed among anaemics. To continue anemia control strategies with reasonable care and diligence was recommended.

## Background

According to the United Nations (UN) estimates, approximately half of pregnant women suffer from anemia worldwide. Anemia prevalences during pregnancy differed from 18% in developed countries to 75% in South Asia [1]. Nutritionally related iron deficiecy is the main cause of anemia throughout the world. It is especially common in women of reproductive age and particularly during pregnancy. The demand for iron increases about six to seven times from early pregnancy to the late pregnancy [2]. Besides poor nutrition, frequent labour, multiparity, abortions, parazitic enfestations, consuming excess tea or coffee after meals determined as the predictors of anemia in reproductive age women [3].

Studies well indicated the association of anemia with maternal morbidity and mortality. Worldwide, anemia contributes to 20% of all maternal deaths [4]. Anemia in pregnancy also lead to premature births, low birth weight, fetal impairment and infant deaths. It reduces the productivity of women. The reduction in women's productivity places an economic burden on the families, communities and the societies [4]. Recently, mental impairment of children who were anaemic in the very begining of their life have been reported. All of those showed the necessity of special control program for anemia in vulnerable population [3-5].

Folate deficiencies since 1960s and B12 vitamin deficiencies since 1990s defined as contributing causes of nutritional anemia [6]. Folate deficiency is common as a result of dietary deficiency or increased demand as in pregnancy. The prevalence of folate deficiency in pregnant patients varies from 1 to 50%. The prevalence is higher among economically deprived patients. B12 vitamin is mostly found in foods of animal origin and deficiency was not reported as a frequent cause of anemia. Anemia due to zinc, copper, vitamin A or other vitamin and minerals are also considered in the literature [3,5].

Since 1994, based on the recommendation of UN, experts in World Health Organization (WHO) and other agencies had developed indexes to monitor and evaluate governments' progress in reproductive health. Prevalence of anemia among pregnant women was one of the key indicators of the indexes [7]. Reproductive Risk Index (RRI) was developed by Population Action International (PAI) which is a Non Govermental Organization. RRI was scored countries in a five likert scale from very high risk to very low risk from the stand point of reproductive health. The Index ranks countries by the prevalence of anemia, from low (less than 20 percent of women affected) to very high (60 percent or more affected). In the PAI report, 2001, prevalence of anemia among pregnant women in Turkey was reported to be "high", with between 40 and 60%. Thus, RRI scored Turkeyscored in “low risk” group but not in “very low risk” group which also existed [8]. The prevalence of anemia in pregnancy was 73.9% according to a nationwide survey conducted in 1974 [9]. Studies conducted in different parts of Turkey after 1995 revealed anemia prevalence between 27% to 88% with an avarage 50.0% [10]. As a problem of public health, anemia was classified as none (less than 5%), mild (5 to 19.9%), moderate (20 to 39.9%) and severe (40% or more) according to the anemia prevalence at the national level by WHO. Anemia prevalence among pregnant women in Turkey was reported as 40.2% in WHO report [11]. PAI and WHO estimates seems to be based on those data sources before 2000s.

Prevalence studies were recomended by WHO both for monitoring the progress in reproductive health and to determine the severity of anemia since national iron supplementation program were planned according to the severity [5,11].

The purpose of this study was to determine the anemia prevalence with the associated factors in Malatya, and to determine the serum iron, folate and B12 vitamin status in anaemic pregnant women.

## Methods

### Study population

The studied population was pregnant women who were resident in Malatya province located in eastern Turkey. According to the 2000 cencus total population of Malatya was 853 658. About fifteen thousand live births were reported to Health Directorate from health institutions every year and most of them (70%) were occured in urban area. According to Turkey Demographic and Health Survey-2008, 76.3% of pregnant women have received antenatal care at least once in the east Anatolia [12].

### Sampling

A stratified multi-stage probability-proportional-to-size cluster sampling methodology was used in selecting the study population. This method has been used for vaccination coverage and still considered acceptable for community based prevalence studies as a fast survey method for developing countries [13,14]. In the first stage of the sampling, Malatya was divided into two stratums as urban and rural. Settlements without a municipality and all villages were recruited as rural area. In Malatya, prenatal care is given by health units. Health units were divided into subpopulations of 2-3 thousands called health houses. Health houses were assigned as clusters (sampling units). The list of all health houses with their populations was obtained from Malatya Health Directorate. Cumulative list of health houses' (seperately in urban and rural areas) populations was created and 30 clusters were selected by systematic sampling method from a random start in each stratum. The target sample size of 900 was allocated to urban and rural areas as 70% and 30% respectively. Selecting nine women per cluster in rural (a total of 270 women) and 21 women in urban settlements (a total of 630) was planned. However, due to absence at home, being reluctant to participate and acces difficulties to two villages, 580 pregnant women in urban and 243 women in rural settlements were interviewed reaching a total of 823 women. Thus, the coverage rate was 91.4%. Completion of the first three months of pregnancy was determined as selection criteria. The pregnant women who complied with the criteria were selected randomly by using the list of pregnancy monitoring cards at health houses.

### Data collection

Permission from the Rector of University, the Governor of Malatya and the Director of Malatya Health Directorate was taken to conduct the field survey. Data collection was performed between November 2003 and May 2004 by two teams with three persons in each team. In rural areas, selected women were visited at their homes in association with midwifes while in urban areas they were invited to the health units. After giving voluntary and informed consent, women were administered a face to face questionnaire including items about socio-demographic, fertility characteristics, diet behavior and anemia history. Questionnaire pretest was conducted on a different pregnant women group before the survey. Finally, a total of 10 ml of venous blood sample was obtained from each participant. Two ml blood was drawn into EDTA for complete blood count (CBC) and 8 ml was drawn into plain glass tubes for use in biochemical analysis.

### Laboratuary analysis

Complete blood count was achieved within four hours with Beckman-Coulter, USA. Iron, folate and B12 vitamin was studied among anaemic women after storing sera at -20°C for six months. Radio immunassay method was used to determine folic acid and vitamin B12 levels (Immulite 2000, Diagnostic Product Corporation, Los Angeles, CA,USA). Serum iron concentration and unsaturated iron binding capacity (UIBC) was measured using Olympus System (Au 2700, Germany).

### Assessment of anemia, iron, iron binding capacity, folic acid and vitamin B12

Cutoff values for Hb and hematocrit concentrations were selected on the basis of gestational age, using WHO criteria. The woman categorized as anaemic if the Hb < 11.0 g/dl. The frequency of the pregnant women with Hb concentrations < 10.5 g/dl at the second trimester was also presented. Manufacturer reference were used to define the normal ranges for mean corpuscular volume (MCV), 80.0-98.0 fl; mean corpuscular hemoglobin (MCH) 27.0-33.5 pg; mean corpuscular hemoglobin concentration (MCHC) 32.0-36.0 g/dL; and erythrocyte width distribution (RDW) 11.9-14.5%. Cut-off level used to indicate serum iron, folic acid and vitamin B12 deficiencies were 50 μg/dl, 3 ng/ml, and 148 pg/ml. respectively according to the literature [15,16]. Transferrin saturation was calculated using the equity of serum iron × 100/TIBC (TIBC = UIBC + serum iron). Transferrin saturation less than 10.2% defined as low serum transferrin saturation and used as the indicator of iron deficiency anemia [17,18].

### Statistical analysis

Data entry and statistical analysis was performed using the SPSSWIN 9.0 program. Descriptive data are presented as percentages or as means± SD. Chi-square test was performed to detect any association between anemia prevalences and independent variables. Mann-Whitney U and Kruskall Wallis tests wereused in making comparisons between the red blood cell indices. A p value of < 0.05 was considered statistically significant. Backward logistic regression analysis was performed to evaluate the independent association existing between the potential risk factors and anemia. Independent variables that were significant at the p = 0.05 level in univariate analysis were included in multivariate analysis to control for confoundings in regression models. Trimester, family income, number of live births, number of living children, family structure, PICA, receving social support were included in regression model as dichotomous variables. The results were presented in odds ratios (OR) and 95% confidence intervals. Hb < 11.0 g/dl was considered anemia for general sample while doing the statistical analysis.

## Results

The average age of the pregnant women was 26.5 ± 0.2 years. The mean ages of anaemic and nonanemic women were similar, 26.9 and 26.4 years respectively (p > 0.05).Of the women, 10.2% were illeterate, 55.1% were primary school graduates. The majority were in the second trimester (63.7%) and 36.3% were in third trimester. Of the participants 33.7% were primipars. The mean number of live births was 1.3 ± 0.05 and the mean number of living children was 1.2 ± 0.05. The average monthly income was 409 ± 11.7 million Turkish Liras (about 272 $), 52.8% had a monthly income under minimum wage (303 million Turkish Liras), 12.9% had received financial support from state and 37.6% had no health insurance. The average monthly income for anemic and nonanemic women was similar (p > 0.05). A total of 29.6% of the women were living in the rural area.

Table 1 shows the mean haematological values and prevalence of anemia by personal characteristics.

**Table 1:** Distribution of mean blood indices and anemia prevalence by personal characteristics

	Mean							
**Personal characteristics**	**MCV (fl)**	**MCH (pg)**	**MCHC (g/dl)**	**RDW(%)**	**Htc%**	**Hb (g/dl)**	**Anemia prevalence^# ^% (n)**	**Total N**

Age								
15-19	84.9	29.1	34.2	14.2	33.6	11.5	24.3(17)	70
20-24	85.5	29.5	34.2	13.9	33.5	11.5	27.6(77)	279
25-29	84.9	29.2	34.3	13.9	33.8	11.6	23.9(56)	234
30-34	85.5	29.3	34.3	14.0	33.5	11.5	30.0(48)	160
35-39	85.5	29.5	34.4	14.2	33.0	11.4	31.8(21)	66
≥ 40	85.0	28.9	33.9	14.6	33.4	11.4	28.6(4)	14

Education								
Illeterate	84.8	29.2	34.3	14.3	33.1	11.4	35.7(30)	84
Literate & primary completed	85.2	29.2	34.2	13.9	33.6	11.5	26.2(129)	492
Secondary school & higher education completed	85.6	29.7	34.3	14.0	33.6	11.5	25.9(64)	247

Family income (million TL)		*^1^					*^1^	
≤ 250	85.4	29.3	34.3	13.8	33.6	11.5	25.8(74)	287
251-500	84.6	29.0	34.2	14.1	33.4	11.4	31.8(114)	358
501-750	86.3	30.6	34.5	13.9	33.8	11.7	17.3(13)	75
751-1000	86.1	29.6	34.3	14.0	34.0	11.7	21.3(16)	75
≥ 1000	86.9	30.0	34.5	14.6	33.9	11.7	21.4(6)	28

Social support							*^1^	
Receiving	85.5	29.4	34.3	13.9	32.9	11.3	35.8(38)	106
Not receiving	85.2	29.3	34.3	14.0	33.7	11.5	25.8(185)	717

Family structure			*^1^	*^1^			*^1^	
Nuclear	85.2	29.4	34.3	14.1	33.5	11.5	29.9(144)	482
Extended	85.4	29.2	34.2	13.9	33.7	11.5	23.2(79)	341

Gestational age	*^1^	*^1^	*^1^		*^1^	*^1^	*^1^	
Second (4-6 months)	85.9	29.7	34.5	13.9	33.8	11.7	21.2(111)	524
Third (7-9 months)	84.2	28.6	33.9	14.2	33.1	11.2	37.5(112)	299

Number of living children							*^1^	
0	85.4	29.4	34.2	14.0	33.9	11.6	25.0(85)	340
1	85.4	29.3	34.2	14.0	33.5	11.5	26.9(61)	227
2	85.0	29.2	34.3	14.0	33.4	11.5	29.0(40)	138
3	85.7	29.7	34.6	13.9	33.7	11.6	18.6(11)	59
≥4	84.2	29.0	34.3	14.1	32.4	11.1	44.1(26)	59

PICA							*^1^	
Positive	82.1	27.9	33.9	14.9	32.7	11.1	37.0(34)	92
Negative	85.7	29.5	34.3	13.9	33.7	11.6	25.9(189)	731

Red meat, poultry, fish consumption								
1 portion everyday	85.5	29.4	34.3	14.1	33.9	11.6	19.4(13)	67
Less frequently	85.2	29.3	34.3	14.0	33.5	11.5	27.8(210)	756

Fruit and vegetables consumption								
1 portion everyday	85.8	29.5	34.3	13.9	33.7	11.6	24.3(84)	346
Less frequently	84.9	29.2	34.2	14.1	33.5	11.5	29.1(139)	477

Drinking tea at breakfast								
Yes	85.2	29.2	34.2	14.0	33.5	11.5	27.7(205)	741
No	86.3	30.6	34.6	14.1	33.9	11.7	22.0(18)	82

Total	85.3	29.3	34.3	14.0	33.6	11.5	27.1	823

* P < 0.05, chi-square test

^1 ^Income levels were categorized according to the Turkish Statistical

Institue "household budget survey"s minimum income in 2004

^# ^Hb < 11.0 g/dl

The mean values for the whole group for MCV, MCH, MCHC, RDW, Htc and Hb were 85.3 fl, 29.3 pg, 34.3 g/dl, 14.0%,33.6% and 11.5 g/dl, respectively while the values for anaemic group were 81.1 fl, 27.5 pg, 33.8 g/dl, 14.8%, 29.9% and 10.1 g/dl, respectively (p < 0.05). All haematological values were lower in anaemic group comparing with the nonanaemic group except the mean RDW value. The mean RDW (14.8%) was significantly higher among anaemic pregnants. As seen in Table 1, observed differences varied as a result of different haematological means as well as different personal characteristics.

Overall, the anemia prevalence was 27.1% among the studied pregnant women. Not many women had severe anemia (Hb concentration was < 7.0 g/dl in one women or < 8.0 g/dl in 3 women).

The proportion of anemia was significantly higher among those women whose monthly family income was less than 500 million TL (29.1%) (p < 0.05). For the subjects who received financial support (35.8%) and lived in nuclear families (30.0%) the occurance rate of anemia was significantly higher (p < 0.05). Anemia was more frequent at the third trimester (37.5%) than at the second (21.2%) (p < 0.05). The proportion of the pregnants with a Hb concentration under 10.5 g/dl at the second trimester was 12.6%.

Of the participants, 278 (33.8%) were primigravidas, 318 were nullipars (38.6%) and 230 (27.9%) were primipars. Anemia prevalences were 25.9%, 24.8% and 27.0% among them, respectively. There was no statistically significant difference between number of pregnancies and anemia prevalence (p>0.05). Anemia prevalence was highest in those with four and more live births (42.3%) and in women with four or more living children (44.1%) (p < 0.05).

There was no significant relationship between the prevalence of anemia and the women' age, educational level and dietary behaviors except soil eating by univariate analysis.

Of the pregnant women, 90.0% drank tea at breakfast, 40.2% consumed egg, 8.1% consumed red meat, poultry or fish and 42.0% consumed fruit and vegetables daily. Soil eating was reported by 11.2%. The percentage of soil eating was higher among illeterate (19.0%) and lower among the group who had graduated from secondary school or had higher education (5.3%) (p < 0.01). Anemia prevalence was higher among those women who ate soil (37.0%) (p < 0.05). Daily animal protein consumption frequency was 4.2% among the low-income women (< 332$), whereas it was 39.3% among high-income women (≥ 332$) (p < 0.05). Similarly, daily fruit and vegetables consumption frequency was more frequent (60.7%) among high-income and less frequent (28.6%) among low-income women (p < 0.05).

Significantly associated variables in univariate analysis were put into backward stepwise logistic regression analysis to determine the independent predictors of anemia at pregnancy (Table 2). After logistic regression analysis, having four or more living children (OR = 2.2), being at third trimester (OR = 2.3) and having a low family income (OR = 1.6) were determined as the independent predictors of anemia during pregnancy.

**Table 2:** The predictors of anemia during pregnancy (logistic regression analysis)

Variables	Odds Ratios	Confidence Interval (95.0%)	B coefficient	P value	N
Number of living children					
< 4	1	-	-	-	764
≥ 4	2.180	1.251-3.797	0.779	0.006	59

Gestational age					
Second trimester	1	-	-	-	524
Third trimester	2.254	1.639-3.099	0.813	0.001	299

Family income					
≥ 500 million TL (≥ 332$)	1	-	-	-	178
< 500 million TL (< 332$)	1.572	1.037-2.383	0.452	0.033	645

-: Reference Category

Percentage distribution of micronutrient deficiencies and mean haematological values among anaemic pregnant women by some personal characteristics were presented in Table 3.

**Table 3:** Frequency of micronutrient deficiences and mean haematological values among anaemic pregnants by some personal characteristics

Personal characteristics	Total N	Micronutrient deficiencies (%)			Mean (±SD) haematological values					
		**İron**	**Folate**	**B12**	**Hb(g/dl)**	**Htc(%)**	**MCV(fl)**	**MCH(pg)**	**MCHC (g/dl)**	**RDW(%)**

Number of living children				*						
0	85	55.3	72.9	29.4	10.1 ± 0.7	30.1 ± 1.8	81.0 ± 8.0	27.3 ± 3.2	33.6 ± 1.1	15.0 ± 2.6
1	61	52.5	67.2	24.6	10.1 ± 0.8	29.8 ± 1.9	81.0 ± 8.2	27.5 ± 3.5	33.8 ± 1.3	14.8 ± 2.4
2	40	60.0	72.5	45.0	10.1 ± 0.7	29.9 ± 2.1	81.3 ± 7.3	27.6 ± 2.9	33.8 ± 0.9	14.6 ± 1.7
3	11	63.6	81.8	54.5	10.1 ± 0.6	29.6 ± 1.1	83.9 ± 10.1	28.7 ± 4.0	34.1 ± 1.0	13.9 ± 1.7
≥4	26	65.4	73.1	50.0	10.1 ± 0.7	29.8 ± 1.8	80.6 ± 7.6	27.4 ± 3.1	33.9 ± 1.0	14.9 ± 1.8

Family income^2 ^(million TL)		*								
≤ 250	74	62.2	71.6	37.8	10.1 ± 0.6	29.8 ± 1.7	82.2 ± 7.4	28.0 ± 3.1	33.9 ± 1.1	14.3 ± 1.9
251-500	114	59.6	75.4	33.3	10.1 ± 0.8	29.9 ± 2.0	79.9 ± 8.1	27.0 ± 3.3	33.7 ± 1.1	15.0 ± 2.5
501-750	13	30.8	69.2	53.8	10.2 ± 0.6	30.6 ± 1.9	81.6 ± 9.1	27.3 ± 3.5	33.7 ± 1.2	15.2 ± 1.9
≥ 751	22	40.9	54.5	18.2	11.7 ± 0.7	34.0 ± 1.9	86.4 ± 7.9	29.7 ± 3.2	34.3 ± 1.2	14.2 ± 2.3

PICA							*	*	*	*
Positive	34	70.6	82.4	41.2	9.8 ± 0.9	29.5 ± 2.4	77.0 ± 7.8	25.8 ± 3.2	33.4 ± 1.1	15.5 ± 2.6
Negative	189	54.5	69.8	33.3	10.2 ± 0.7	30.0 ± 1.7	81.9 ± 7.8	27.9 ± 3.2	33.8 ± 1.1	14.7 ± 2.1

Drinking tea at breakfast			*							
Yes	205	58.0	74.1	35.6	10.1 ± 0.7	29.9 ± 1.8	81.0 ± 7.8	27.4 ± 3.2	33.8 ± 1.1	14.8 ± 2.3
No	18	44.4	44.4	22.2	10.1 ± 0.8	30.0 ± 2.3	82.6 ± 9.3	28.0 ± 3.9	33.8 ± 1.2	14.9 ± 1.9

Red meat, poultry, fish consumption										
1 portion everyday	146	93.8	96.8	92.5	10.1 ± 0.8	30.0 ± 1.6	81.3 ± 5.9	27.2 ± 2.7	33.5 ± 1.5	15.6 ± 2.8
Less frequently	77	94.5	93.1	97.4	10.1 ± 0.7	29.9 ± 1.9	81.1 ± 8.1	27.5 ± 3.3	33.8 ± 1.1	14.7 ± 2.2

Fruit & vegetables consumption										
1 portion everyday	63	57.3	66.7	65.1	10.2 ± 0.7	30.0 ± 1.8	82.2 ± 7.0	27.9 ± 3.0	33.9 ± 1.1	14.7 ± 1.8
Less frequently	160	66.1	60.6	57.1	10.1 ± 0.7	29.9 ± 1.9	80.5 ± 8.4	27.2 ± 3.4	33.7 ± 1.1	14.9 ± 2.5

Current use of iron treatment			*							*
Yes	47	46.8	42.6	29.8	10.1 ± 0.9	29.7 ± 2.1	80.0 ± 9.2	27.2 ± 3.8	33.9 ± 1.2	15.5 ± 2.4
No	176	59.7	79.5	35.8	10.1 ± 0.7	30.0 ± 1.8	81.5 ± 7.6	27.6 ± 3.1	33.7 ± 1.1	14.6 ± 2.2

Total	223	57.0	71.7	34.5	10.1 ± 0.7	29.9 ± 1.9	81.1 ± 7.9	27.5 ± 3.3	33.8 ± 1.1	14.8 ± 2.3

^2 ^Family income category reduced in four groups since the number in the fifth group was small, * P < 0.05, Chi-square, Mann-Whitney U test, Kruskall Wallis tests

Of the anaemic women, 12.6% had UIBC level >300 μg/dl, 68.2% had TIBC level >480 μg/dl, 50.2% had a transferrin saturation less than 10.2% (data not shown) and 57.0% had serum Fe level <50 μg/L (Table 3). Folate and B12 vitamin deficiencies were found in 71.7% and 34.5% of the anaemic women, respectively. Only a small number (11) of participants had serum B12 level <100 pg/dl (4.9%). The proportion of folate deficiency was significantly higher among women who drank tea at breakfast (74.1%) and significantly lower among women who were under iron medication (42.6%) (p < 0.05). B12 vitamin deficiency was more prevalent among women with more than two living children and iron deficiency was more prevalent among women with a monthly family income less than 500 million TL (<332$) (p < 0.05). The proportion of folate deficiency was 72.9% among anaemic nullipars (Table 3) and 72.2% among anaemic primigravidas (data not shown).

In analyzing the red blood cell count, significant differences were observed in the mean values for MCV, MCH, MCHC and RDW by soil eating and in the mean value for RDW by current use of iron treatment (p < 0.05). The mean RDW value was higher among women with PICA (15.5%) and among those women who were under iron medication (15.5%). The mean value for MCV was close to the lower limit (81.1 fl) among anaemics (minimum: 57.9, maximum: 101.5). Among women with three children the mean value for MCV was 83.9 ± 10.1 fl and, the proportions of B12 vitamin and folate deficiencies were also higher in this category indicating the presence of mixed anemia (Table 3).

Of the anaemic pregnants, 38.1% had a microcytic-hypochromic anemia (MCV < 80 fl & MCH < 27 pg), 56.5% had a normocytic-normochromic (MCV and MCH within normal range), 0.9% had a macrocytic (MCV > 98.0 fl) anemia and 4.5% had combined morphologic type of anemia. Iron deficiency was the most prevalent micronutrient deficiency (67.1%) among microcytic-hypochromic anemia (p < 0.05) and, folat deficiency was common in all morphologic types (p > 0.05) (Table 4).

**Table 4:** Distribution of micronutrient deficiencies by morphologic type of anemia

Morphologic type of anemia	Micronutrient deficiencies			Total
	Iron	Folat	B12 vitamin	
	**n(%)**	**n(%)**	**n(%)**	**n(%)**

Microcytic-hypochromic anemia (MCV < 80 MCH < 27)	57 (67.1)*	66(77.6)	27(31.8)	85 (38.1)

Normocytic-normochromic anemia (MCV and MCH within normal range)	65(51.6)	87(69.0)	47 (37.3)	126(56.5)

Macrocytic (MCV>98)	1 (50.0)	1 (50.0)	0 (0.0)	2 (0.9)

Others (Kombined morphology)	4(40.0)	6(60.0)	3 (30.0)	10(4.5)

Total	127 (57.0)	160(71.7)	77(34.5)	223(100.0)

* p < 0.05, chi-square test

Overall, 27.1% of the pregnant women were under iron medication and 10.9% were taking both iron and folic acide supplements. Of the women, 52.9% had received iron therapy at least once in their lifetime. Being under iron medication was significantly different between family income categories. Of the pregnant women in the highest income category, 71.4% were under iron medication while the proportion was only 13.9% in the lowest income category (p < 0.05). Of the anaemic pregnants, 21.1% took iron supplements, 11.7% took both folate and iron supplements and, 6.3% vitamin tablets. Of the subjects with a monthly family income < 500 millionTL, 18.1% took iron supplements while 37.1% of the subjects in higher income group took iron supplements (p < 0.05). Women with no health insurance were less likely (15.7%) than the insured to receive iron supplements (p < 0.05).

## Discussion

The study conducted in Malatya, which is an eastern Anatolian province with 800 000 inhabitants, showed a moderate prevalence of anemia (27.1%). Our findings did not correlate with the results reported by PAI and WHO which indicated that pregnant women in Turkey have had severe anemia [8,11]. Our results also notice that anemia trends might have changed in Turkey and a nationwide anemia study is necessary for determining the new anemia status and for international notification. As a matter of fact, the reduction in anemia prevalence from that projected earlier in 1970s is expectable since there was a rapid socio-economic growth of the Turkish population over the past two-three decades which had a major impact on both health status and disease patterns throughout the country and particularly in east Anatolia. Since 1985, governments in colloboration with international organizations (UNICEF) conducted programs related to mother and child health such as safe motherhood, antenatal care, nutrition education of the public and food aid to the the low income families. Human resouces, education, industry, agriculture and health sector was improved through national development plans and, Eastern Anatolian Project including Malatya since 1997 [19-21].

Even though the study showed 27.1% prevalence, this is higher than the prevalences in European countries (25.1%) and in Americas (24.1%) or averaging 18% in developed countries [5,11]. Therefore, it is necessary to continue anemia control programs.

Anemia prevalence in our study was also lower than those reported from different parts of the country such as 29.4% in Afyon [22] and 42.4% in Elaziğ provinces, the latter is in the eastern Anatolia near Malatya [23].

Socio-economic status is a known determinant of anemia. In this study, anemia was more prevalant among those women who received financial support (p < 0.05) and who had a low monthly family income (OR = 1.6). The majority of anaemic pregnants lived in nuclear rather than extended families which might seem paradoxical. As a result of industrialization, urbanization and internal migration, people are more likely to be living in nuclear households in Turkey, recently [19,21]. However they might be low income families that immigrated for a better life. Family economics and nutrition related problems need to be investigated in detail in such households to determine the contributing effects of social structure and life styles on morbidity. Anemia prevalence was 2.3 times more prevalant at third trimester; The 21.2% prevalence of anemia at the second trimester increased to 37.5% at the third trimester comparable to the literature [24-26]. This might be due to hemodilution but, it might also indicate poor prenatal care.

Anemia was 2.2 (OR) times more prevalent in women with four or more living children than in women with fewer children. Multiparity may induce anemia by reducing maternal iron reserves at every pregnancy and by causing blood loss at each delivery. The mean number of living children was 1.2 in our study. It was reduced to 2.4 from 2.7 between 1993 and 2003 due to the implementation of family planning services and safe motherhood programs countrywide after 1980s [19,27]. Effective implementation of these programs in Malatya might be reason for the low anemia prevalence in our study from that reported in 1970s. However, we did not observe a homogeneous increase in anemia prevalence with the increase in number of living children by univariate analysis (Table 1). About one fourth of primigravidas, nullipars and primipars were anaemic in our study. The non-homogeneity with respect to parity was seemed to be a result of high prevalence of folate deficiency among anaemic women (71.7%).

The study showed that one pregnant women out of ten was eating soil and anemia was more prevalant among soil eaters (37.0%). Soil eating (PICA) is known to be an old problem among Turkish women and it is common in many underdeveloped countries [28]. İt is still debate whether soil eating causes anemia or anemia leads soil eating. Zinc or other mineral deficiencies may contribute to PICA [29,30]. The frequencies of iron, folate, and B12 vitamin deficiencies among women with PICA were 70.6%, 82.4% and 41.2%, respectively. Thus, questioning PICA at prenatal visits and laboratory examination of pregnant women with PICA is recommended.

It was stated in the literature that tea consumption and low intake of red meat were associated with anemia [31-33]. Some studies emphasized that tea reduces iron absorbtion but does not influence iron status in people with adequate iron stores [34-37]. Meat is a good source of high quality protein, iron and zinc and of all the B-vitamins except folic acid. Meat consumption was reported to be 21 kg/capita/year for Turkey, 124 kg/capita/year for USA and about 100kg/capita/year for European countries [38]. These data might explain the lower anemia prevalences among those developed countries. We found that 90% of pregnants drank tea at breakfast and only 8% consumed animal protein daily. Our study did not reveal a significant association with tea and meat consumption though anemia was less prevalent in women who consumed one portion of animal protein daily (19.4%) and who did not drink tea at breakfast (22.0%). Consuming tea between meals and simultaneously consuming vitamin C and/or meat, fish and poultry were the main dietary recommendations to prevent anemia [37]. A recent publication reported that green and black tea had the potantial risk of diminished folic acid bioavailability [39]. Drinking tea at breakfast and just after the meal are common unhealthy dietary habits in Turkey. Thus, continous nutrition education and monitoring programs should be developed at all levels according to the recommendations to combat anemia.

Among the anaemic women, half had a transferin saturation less than 10% indicating iron deficiency, one third (34.5%) were deficient in B12 vitamin and more than two third (71.7%) were deficient in folate. Pehlivanoğlu and Koç reported higher percentages of iron deficiencies among anaemic pregnant women in İstanbul; 68.5% and 62%, respectively [40,41]. High percentages of pregnants were at risk for deficiencies of vitamin B12 (48.8%) and folate (59.7%) in early pregnancy and vitamin B12 (80.9%) and folate (76.4%) during late pregnancy in Açkurt's study [10]. Koc et al. reported higher B12 deficiency (48%) and lower folic acid deficiency (12%) than ours [41]. All these studies were conducted at clinics, and so to determine the contribution of vitamin deficiencies to anemia a population based survey is necessary. İron deficiency defined as the most frequent form of the nutritional anemia in pregnant women in the literature followed by folate and B12 vitamin deficiencies [3,6,24,42]. Folate deficiency was more frequent than iron deficiency in our study. In a study conducted in a western city in Turkey, serum folic acid levels were found to be marginal for 46% (3 to 5.9ng/mL) and at deficient levels for 16.3% (< 3ng/mL) among adolescent girls [43]. Our study showed high prevalences of folate deficiency even in primigravidas and nullipars raised doubt if there was a common folate deficiency problem in the population. The prevalence of neural tube defects (NTDs) in Turkey has been reported to be 30.1 per 10000 births [44]. In order to decrease births affected by NTD, the fortification of all enriched grain products with folic acid have been implemented in USA since 1998 and folate deficiency in population was reduced. Further studies recommended to determine the folate deficiency burden in the general population and to decide if fortification of appropiate foods like wheat flour which is the main staple food of the country with folic acid or with other micronutrients as tried in USA, Canada and Chile [45].

We found that, most of the anemias were normocytic-normochromic (56.5%) and, 38.1% were microcytic-hypochromic. Despite the high propotions of folate deficiency in anaemic women, only 2 cases (0.9%) were macrocytic. It was stated in the literature that folate and B12 vitamin deficiencies caused macrocytic anemia, however concurrent presence of iron deficiency resulted in normocytic anemia [3]. The mean haematological values for the anaemic women in our study confirmed this inference (Table 3). In anaemic women, iron and folate deficiencies were both common, mean MCV value was close to the lower limit, mean Hb value was 10.1g/dl, and RDW was 14.8% showing that there was a mixed anemia with the dominant cause being iron deficiency [46].

The limitations of the study should be emphasized particularly in relation with serum iron, B12 vitamin and serum folate. Fasting or nonfasting status of women was not questioned at drawning of blood samples which the results might have altered. They were studied only in anaemic women so that any comparison with non-anaemic women could not be done. Serum transferrin and red blood cell folate are better indicators of iron and folate deficiency which our budget could not afford. For the serum B12, we could comment that all forms of vitamin B12 in the serum might be detected so the deficiency was high. Also it was observed that serum B12 vitamin cutoff levels for pregnant women were not certain in the literature and being under iron medication might inluence the mean blood indices. Enfections were not questioned either.

In conclusion there was a moderate anemia problem both in the second and third trimesters among pregnant women in Malatya, Turkey. The main predictors of anemia were low family income, being in the third trimester and having four or more living children. Of the anaemic women half was iron deficient, one third was B12 vitamin and two third was folate deficient. The most frequent morphologic type of anemia was normocytic-normochromic anemia and, the complete blood count suggested that anemia was caused by mixed micronutrient deficiency. Only one fifth of the anaemic women were under iron medication. Ten percent of the women were eating soil and anemia was more prevalent among soil eaters. PICA was more prevalent among low-income anaemic women, whereas consuming animal protein daily and being under iron medication were less frequent in low-income anaemic women.

World health Organization recommended supplementation of all pregnant women with a daily dose of 60 mg iron and 400 g folate to control iron deficiency anemia as a primary prevention method [47]. Disadvantages of iron supplementation include poor compliance with treatment, adverse gastrointestinal effects, low absorbsition of iron supplements due to taken with meals or with other minerals but not seperately. However, it was shown that daily iron supplements reduce low birth weight incidence. It is suggested that this reduction leads decrease in chronic diseases and could reduce health care costs [48]. B12 vitamin deficiency association with hematological and neurological problems in infants and chronic diseases in adults including thromboembolic conditions have been reported in the literature [49,50]. Due to that, we recommended daily iron supplementation program with the supplementation of folate and B12 vitamine. Turkey had begun routine iron supplementation to the pregnant women all over the country in 2005 after the survey [51]. The program should be given attention because there is a possibility of neglecting the appropiate and comprehensive examination and therapy of anaemic women due to iron supplement given. Therefore, educating the health personnel on the subject is necessary. Some developed countries supply food rich in iron, calcium, protein and vitamins to the pregnant women. Sweden and USA are among the developed countries conducted such programs [14,52,53]. Access to the low-income anaemic pregnant women and food aid programs towards them need to be considered as immediate recipe in Turkey.

## Conclusions

There was a moderate anemia problem in pregnant women in Malatya, Turkey (27.1%). One fourth of women enter pregnancy with anemia. Low family income (< 332$), gestational age (third trimester) and multiparity were the main predictors of anemia. The main morphologic type of anemia was normocytic-normochromic. Coexisting of iron, folate and B vitamin deficiencies was observed among anaemics. Continuing of daily iron supplementation program with folate supplementation in the beginning of pregnancy and food aid programs towards low-income women was recommended.

## Competing interests

The authors declare that they have no competing interests.

## Authors' contributions

LK participated in the design of the study, perfomed the data collection, performed the statistical analysis and served as the lead author of the manuscript. EP participated in the design of the study, helped to draft the manuscript. ME participated in the design of the study and helped performing the statistical analysis. CD helped to collect data. GG participated in drafting the manuscript. MEF participated in drafting the manuscript. IT carried out the immunoassays and complete blood count. All authors read and approved the final manuscript.

## Pre-publication history

The pre-publication history for this paper can be accessed here:

http://www.biomedcentral.com/1471-2458/10/329/prepub
